# Singing Proficiency of Members of a Choir Formed by Prelingually Deafened Children With Cochlear Implants

**DOI:** 10.1044/2019_JSLHR-H-18-0385

**Published:** 2019-04-25

**Authors:** Jing Yang, Qi Liang, Haotong Chen, Yanjun Liu, Li Xu

**Affiliations:** a Department of Communication Sciences and Disorders, University of Wisconsin–Milwaukee; b Aier Times Ltd., Beijing, China; c Department of Communication Sciences and Disorders, Ohio University, Athens

## Abstract

**Purpose:**

A group of 10 prelingually deafened children with cochlear implants (CIs) formed a choir and received 21 months of formal music training. The purpose of this study was to evaluate the singing proficiency of these children.

**Method:**

The participants included all choir members (7 girls and 3 boys, mean age of 9.5 years old) who were unilateral CI users. Meanwhile, 8 age-matched children with normal hearing were recruited as controls and were trained on 1 song for 2 weeks. Individual singing samples without instrument accompaniment were recorded from all participants. The singing samples were subject to acoustic analysis in which the fundamental frequency (F0) of each note was extracted and the duration was measured. Five metrics were developed and computed to quantify the accuracy of their pitch and rhythm performance. The 5 metrics included (a) percent correct of F0 contour direction of adjacent notes, (b) mean deviation of the normalized F0 across the notes, (c) mean deviation of the pitch intervals, (d) mean deviation of adjacent note duration ratio, and (e) mean absolute deviation of note duration.

**Results:**

The choir members with CIs demonstrated high accuracy in both pitch and tempo measures and performed on par with the children with normal hearing. Early start of music training after implantation and use of bimodal hearing contributed to the development of better music ability in these children with CIs.

**Conclusion:**

These findings indicated that rigorous music training could facilitate high singing proficiency in prelingually deafened children with CIs.

Music, like human language, is one of the most complicated, demanding, yet unique human activities. Music events, including both instrumental and vocal performance, use the basic elements of pitch, rhythm, loudness, and timbre to convey information. Vocal music performance, simply called *singing*, is universal to all cultures and regions. Singing plays important communicative, social, and religious roles throughout human history. For people with hearing impairments, due to the damaged peripheral or central auditory system, their music processing and vocal singing show varying degrees of deficits (Mao et al., 2013; B. C. J. Moore & Carlyon, 2005; Xu et al., 2009).

## Pitch Perception and Production in Cochlear Implant Users

Contemporary multichannel cochlear implant (CI) is a hearing prosthesis that bypasses the injured part of the inner ear and directly stimulates the auditory neurons electrically. This device conveys adequate information for speech recognition and precise rhythmic information for music perception, which helps people with severe-to-profound sensorineural hearing loss restore partial auditory sensation and greatly improves their communicative abilities (Wilson & Dorman, 2008). However, because pitch information is not explicitly encoded in CI stimulations, pitch-related tasks in music and speech (e.g., lexical tone, intonation, voice, and emotion recognition) are still challenging for CI users (Chatterjee, Sis, Damm, & Kulkarni, 2018; Hopyan-Misakyan, Gordon, Dennis, & Papsin, 2009; Limb & Rubinstein, 2012; Wang, Zhou, & Xu, 2011; Wilson, 2015). So far, many studies have documented the deficits in tone perception and production in prelingually deafened children with CIs (Y. Chen & Wong, 2017; Ciocca, Francis, Aisha, & Wong, 2002; Han et al., 2007; Liu, Peng, Zhao, & Ni, 2017; Mao & Xu, 2017; Peng, Weiss, Cheung, & Lin, 2004; Tan, Dowell, & Vogel, 2016; Xu et al., 2004, 2011; Xu & Zhou, 2011). In studies evaluating music perception in CI recipients, researchers have found that, although CI users perform relatively well on rhythm perception such as rhythm pattern recognition and rhythm discrimination, they show evident deficiency in pitch-related tasks such as pitch discrimination, pitch ranking, melody contour identification, and so forth (Cooper, Tobey, & Loizou, 2008; Galvin, Fu, & Nogaki, 2007; Gfeller et al., 2007; Limb & Rubinstein, 2012; McDermott, 2004; Scorpecci et al., 2012). Compared to adults with normal hearing (NH) who can discriminate two pitches that are one semitone apart, pitch discrimination threshold in CI users reaches as high as 24 semitones with the average thresholds between three and six semitones (Fujita & Ito, 1999; Gfeller et al., 2002; Kang et al., 2009; See, Driscoll, Gfeller, Kliethermes, & Oleson, 2013; Wang et al., 2011). Given the association between perception and production, poor musical production in children with hearing impairment with CIs has been documented in several previous studies. Nakata, Trehub, Mitani, and Kanda (2006) compared the vocal pitch patterns in a group of 4- to 10-year-old congenitally deafened children with CIs with those of children with NH in the similar age range. The authors found that children with CIs showed mismatched vocal pitch patterns from the target songs but age-appropriate rhythm reproduction as compared to the controls with NH. Xu et al. (2009) recruited seven 5- to 12-year-old prelingually deafened children with CIs and compared their singing performance with that of 14 peers with NH. They developed five metrics to capture the pitch and rhythm aspects of vocal singing accuracy. Together with a later study with additional 37 prelingually deafened children with CIs, 31 children with hearing impairment with hearing aids (HAs), and 37 children with NH (Mao et al., 2013), Xu et al. reported that children with hearing impairment using either CIs or HAs showed significantly poorer performance on pitch-related measures in comparison to the controls with NH. These findings suggest that children with hearing impairment, regardless of being fitted with CI or HA devices, are less likely to achieve satisfactory proficiency in vocal singing. The above mentioned studies suggest that music development in children with hearing impairment is quite disappointing. Nonetheless, one should keep in mind that, although deaf children with CIs showed erroneous tunes in vocal singing, they clearly showed comparable energy, vitality, and joy in singing to their hearing peers (Trehub, Vongpaisal, & Nakata, 2009).

## Story of “Little Dolphins”

The pessimistic view of poor vocal singing ability of children with CIs has been challenged recently because of the success of a group of prelingually deafened children with CIs. In November 2014, a children's choir, called *Tianjin Dolphin Hearing Disabled Children's Choir*, was founded in Tianjin, China. All 10 members (called *Little Dolphins*; seven girls and three boys, aged 5–10 years old) were prelingually deafened children who received cochlear implantation at the age of 1.0–6.5 years old. By the time of being recruited for this study, these children had taken part in weekly singing training for a period of 21 months. Details of the training paradigm are provided in the Method section. In July 2016, the choir participated in the Ninth World Choir Games in Sochi, Russia, and competed with other choirs that were formed with people with NH from countries all over the world. Little Dolphins won a Silver Diploma in the Category of Scenic Folklore in the Open Competition (Interkultur, 2016). In July 2017, the choir participated in the Tenth International Johannes Brahms Choir Festival and Competition in Wernigerode, Germany, and won a Silver Diploma in the Category of Folklore (Interkultur, 2017). The success story of Little Dolphins evoked our strong interest and made us rethink the singing proficiency that children with CIs could reach after prolonged music training.

## Music Training on Pitch Perception in Children With CIs

Previous studies have indicated that music training or music activities are beneficial to children with CIs (Gfeller, 2016). Gfeller, Witt, Spencer, Stordahl, and Tomblin (1998) conducted a survey study examining the involvement and enjoyment of music in children with CIs. Their data showed that a high percentage of tested children with CIs were engaged in formal or informal musical activities and showed increased interest to music postimplantation. More recently, there has been an increasing interest in the effects of music training on speech and music perception in CI users (Abdi, Khalessi, Khorsandi, & Gholami, 2001; J. K.-C. Chen et al., 2010; Cheng et al., 2018; Fu, Galvin, Wang, & Wu, 2015; Innes-Brown, Marozeau, Storey, & Blamey, 2013; Torppa et al., 2014; Yucel, Sennaroglu, & Belgin, 2009). For example, J. K.-C. Chen et al. (2010) examined the perception of a set of two consecutive piano notes ranging from C (256 Hz) to B (495 Hz) in 27 congenitally/prelingually deafened children with CIs. Thirteen of them were involved in formal musical training for a period ranging from 2 to 36 months. The results showed positive correlation between the length of musical training and the accuracy rate of pitch perception. Torppa et al. (2014) tested a number of tasks with regard to fundamental frequency (F0) discrimination, stress perception, prosodic perception, and auditory working memory in 21 children with CIs and the same number of controls with NH. The children with CIs were divided into two subgroups, with one receiving musical training and the other one not receiving training. The authors found positive correlation between musical training and the perceptual performance. Fu et al. (2015) assessed the benefit of musical training on music pitch perception in 14 Mandarin-speaking pediatric CI users. All participants completed a 10-week music training. Melodic contour identification was tested at the baseline condition (0 week) and fourth, eighth, and 10th week of training, and follow-up evaluation was conducted at 1, 2, 4, and 8 weeks posttraining. The results showed that the melodic contour identification score was generally poor at the baseline condition but greatly improved after only 4 weeks of training. Moreover, the follow-up assessment suggested that the performance gains could be well retained till 8 weeks posttraining.

## This Study

Taken together, these studies provided promising results supporting the beneficial effects of music training on pitch-related perceptual performance. Intuitively, the improved pitch perception after music training would suggest improved pitch-related singing performance in CI users. Although there has been some early efforts examining the relationship between music training and auditory learning in CI recipients (Gfeller, 2016; Looi, Gfeller, & Driscoll, 2012), no scientific report has been available on the effects of music training on vocal singing in CI recipients. “Little Dolphins” provided a good opportunity to evaluate singing proficiency of pediatric CI users after a relatively long period of intensive singing training. In this study, we conducted comprehensive acoustic analyses to quantify different aspects of singing performance for all choir members and compared their singing performance with that of age-matched children with NH and previously published data on singing proficiency of pediatric CI users.

## Method

### Participants

Ten prelingually deafened children with CIs (seven girls and three boys) participated in this study. All participants were diagnosed with profound sensorineural hearing loss at birth with the exception of one participant whose hearing loss was diagnosed at the age of 6 years. These participants aged between 7.4 and 12.3 years (*M* = 9.53, *SD* = 1.59), and all received unilateral CIs. The demographic information of these children is listed in Table 1. The age of implantation varied from 1.1 to 6.5 years, and all had at least 2 years of experience with their devices. Five of them wore HAs at the contralateral side, and the other five did not. They all used oral communication, and none of them were identified with other neurological impairment. All participants with CIs attended the “Tianjin Dolphin Hearing Disabled Children's Choir” established in November 2014 in Tianjin, China. Because the long-term music training required high motivation and great commitment of the family with regard to the traveling and time investment, convenience sampling instead of pretraining performance on singing or speech was used as the inclusion criterion for this choir. None of the subjects with CIs had enrolled in any formal musical training prior to their participation in this choir.

**Table 1.** Demographic information of the 10 Little Dolphins choir members with cochlear implants.

Subject no.	Age (years)	Sex	Etiology	Age at implantation (years)	Singing training started (years postimplantation)	Duration of implant use (years)	Implant side	Implant device	Speech processing strategy	Contralateral hearing aid use
1	7.37	F	LVAS	2.38	3.23	4.99	R	1	A	y
2	7.78	F	Unknown	3.11	2.90	4.67	R	1	B	y
3	10.17	M	LVAS	3.89	4.51	6.28	R	1	B	y
4	8.69	F	LVAS	6.50	0.43	2.19	R	2	B	y
5	8.25	M	LVAS	4.84	1.64	3.41	R	1	A	y
6	10.61	F	Genetic	1.12	7.73	9.49	L	1	B	n
7	8.72	F	Unknown	1.35	5.61	7.37	R	1	B	n
8	11.21	F	Unknown	2.12	7.33	9.09	R	1	B	n
9	10.28	F	Unknown	1.94	6.58	8.34	L	1	B	n
10	12.21	M	Unknown	1.00	9.44	11.21	R	1	B	n
*M*	9.53			2.82	4.94	6.70				
*SD*	1.59			1.79	2.89	2.89				

*Note.* F= female; M = male; LVAS = large vestibular aqueduct syndrome; R = right; L = left; Implant device: 1 = Advanced Bionics HiRes 90K cochlear implant with HiFocus 1j electrode; 2 = Advanced Bionics HiRes 90K cochlear implant with HiFocus Helix electrode; Speech processing strategy: A = HiRes-P/Fidelity 120; B = HiRes-P/Fidelity 120 + ClearVoice; y = yes; n = no.

Eight typically developing, Mandarin-speaking children with NH (seven girls and one boy, 6–10 years old with an average age of 8.13 years) were recruited from the Beijing area to serve as the control group. All children with NH had regular music class (typically 1 hr per week) in school. Five of the children with NH had attended weekly private piano lessons out of school for 2–3 years. None of the children with NH received formal vocal singing training. The use of human subjects was reviewed and approved by the Institutional Review Board of Ohio University. All research was performed in accordance with relevant guidelines and regulations, and the informed consent was obtained from a parent of each participant.

### Musical and Speech Training

Prior to the participation in the choir, all children with CIs, except one, had received at least 1 year of postimplantation speech-language rehabilitation at various professional rehabilitation centers in Tianjin. Upon the point when the study was conducted, all participants with CIs had gone through a 21-month musical training. During this period, all choir members had 3 hr of group-based formal music training conducted at the training center every week and 1 hr of individual speech training conducted at home every day. The formal music training was provided by music teachers, and the speech training was provided by caregivers. In addition, each participant was required to have informal practice at home every day, with a total of approximately 8 hr per week, which was under the supervision of the children's caregivers with the assistance of online live tutorial provided by music-majored undergraduate students who served as volunteers for this choir. The content of formal training focused on musical scale for the most part of the first year. Once the children could sing the music scale more or less correctly, they started training on popular children's songs (such as *Twinkle Twinkle Little Star*, *Frère Jacques*, etc.) and songs composed specifically for the choir (such as *Little Carp Jumping the Dragon Gate*, *I Can So You Can*, etc.). The song *Little Carp Jumping the Dragon Gate* was the one that the choir performed in both the Ninth World Choir Games in Sochi, Russia, and the 10th International Johannes Brahms Choir Festival and Competition in Wernigerode, Germany.

For the controls with NH, no specific speech training was provided. Because the song *Little Carp Jumping the Dragon Gate* was composed specifically for the Little Dolphins, it was unfamiliar to the children with NH. They were all trained individually on this song for 2 weeks. For each participant with NH in each week, the training session was supervised by a music teacher and lasted approximately 2 hr. At the end of the 2-week training session, their singing performance of the newly learned song was evaluated by the music teacher and was deemed accurate and satisfactory.

### Data Collection and Analysis

Vocal singing samples were collected individually for both children with CIs and children with NH in a quiet room. Each child was asked to sing the song *Little Carp Jumping the Dragon Gate* without instrumental accompaniment. The singing samples were recorded via a Zoom H4 recorder with a sampling rate of 44.1 kHz. The microphone was placed at approximately 10 cm away from the participants' lips. The recorded samples were transferred to a computer hard disk for further analysis.

As the target population of this study was children with CIs who experience apparent difficulty in pitch-related tasks due to the inherent deficits of pitch coding in the CI devices, acoustic measurements were developed to mainly evaluate the pitch accuracy of the singing. Meanwhile, as tempo is another salient characteristic defining the nature of music, the tempo accuracy was also of particular interest in this study. The target song *Little Carp Jumping the Dragon Gate* had a total of 340 notes. The first 96 notes, which represented the main melodic structure of the song, were isolated for the following acoustic analyses. For each note, the duration was measured, and F0s from the steady portion of articulated vowels were extracted using an autocorrelation algorithm implemented in a custom MATLAB program. The mean F0 of each note was used as the pitch for that note. The F0 values of all 96 notes were converted into semitones using the formula: semitone = 12 log_2_ (F0/261.6). Because each individual participant might sing at various pitch height, we normalized the pitch data in semitones against the mean across all 96 notes. A similar procedure was also applied to the target notes of the music score so that the pitch contour of the sung notes could be optimally aligned with that of the target notes from the music score.

On the basis of the normalized F0s, a number of pitch-based metrics were computed to quantify the singing accuracy of each participant (Mao et al., 2013; Xu et al., 2009). The first metric was percent correct of F0 contour direction of the adjacent notes. The music score of the target song was used as the reference. The direction of any two adjacent notes for each participant was compared with the direction of the same adjacent notes in the target music score. If the direction was consistent with that in the target score, it was counted as correct. The identical adjacent notes with no pitch change in the target music score were ignored and not counted. For each participant, the percent correct of F0 contour direction of adjacent notes was then calculated by dividing the total number of correct pitch changes by the total number of pitch changes in the target music score. The second metric was the mean deviation of the normalized F0 across the notes. This measure was derived by calculating the absolute difference between each participant's individual notes and the corresponding target notes from the music score. Then, the absolute differences were averaged across all 96 notes for each participant. The third metric was mean deviation of the pitch intervals. To obtain this measure, we first calculated the pitch intervals of any two consecutive notes of the target notes in the music score. Then, the pitch intervals of corresponding two consecutive notes of the sung notes by each participant were calculated. Finally, the absolute differences of the pitch intervals between the song by each participant and the target music score were calculated and averaged.

Two additional metrics were derived to quantify the tempo accuracy of the singing based on the note duration data. One of these metrics was the mean deviation of adjacent note duration ratio. To calculate this measure, the duration ratios of any two adjacent notes were calculated for the target music score. The duration ratios of corresponding two adjacent notes in each participant were also calculated. Then, the absolute differences of the duration ratios between each participant and the target music score were calculated and averaged. This metric provided an accuracy measurement of relative tempo of the songs. The other measure was the mean absolute deviation of note duration, which was calculated by averaging the absolute differences of note duration between each participant and the target music score for all 96 notes. This metric provided an accuracy measurement of absolute tempo of the songs. Previous literature have shown that casual singers in normal population usually sing with a faster tempo than trained singers (Dalla Bella, Giguère, & Peretz, 2007). As temporal information is well maintained and delivered through CI devices, the measure of mean absolute duration deviations enabled us to examine whether the children with CIs demonstrated improvement in tempo feature after intensive music training.

## Results

**Figure 1.**
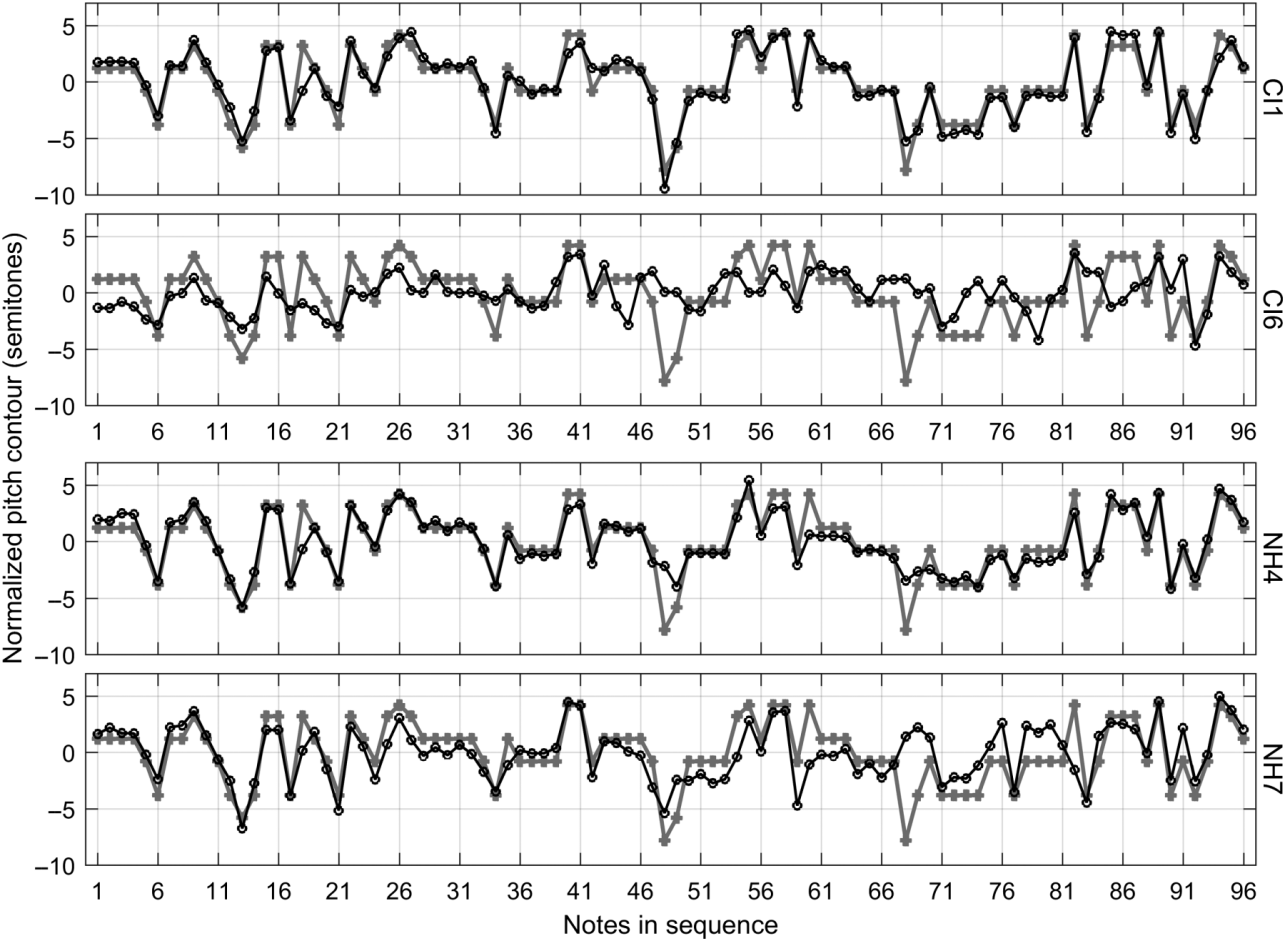
Normalized pitch contours of two children with cochlear implants (CI; top two panels, subjects CI1 and CI6) and two children with normal hearing (NH; bottom two panels, subjects NH4 and NH7). The first 96 notes of the song *Little Carp Jumping the Dragon Gate* were presented here. For each subject (each panel), the gray lines and filled symbols represent the target song, whereas the black lines and open symbols represent the pitch contour of the song produced by the subject. Both the target song and the recorded song were normalized to their respective means in semitone.

Figure 1 displays the normalized pitch contours of two children with CIs and two children with NH. The two children with CIs (CI1 and CI6) were the best and the poorest performer based on the mean note deviation and mean interval deviation of the pitch contours (see Method section for details on these two metrics) among the CI group. Likewise, the two children with NH (NH4 and NH7) were the best and the poorest performer among the NH group. The pitch contours of the best performers in both CI and NH groups aligned very well with the target pitch contour. By contrast, the pitch contours of the poorest performers did not align well with the target pitch contour, showing large deviations (e.g., second half of NH7) and compressed pitch range relative to the target pitch contour (e.g., CI6). The pitch contours of other subjects, not shown here, laid in between these two extreme cases for the respective groups.

**Figure 2.**
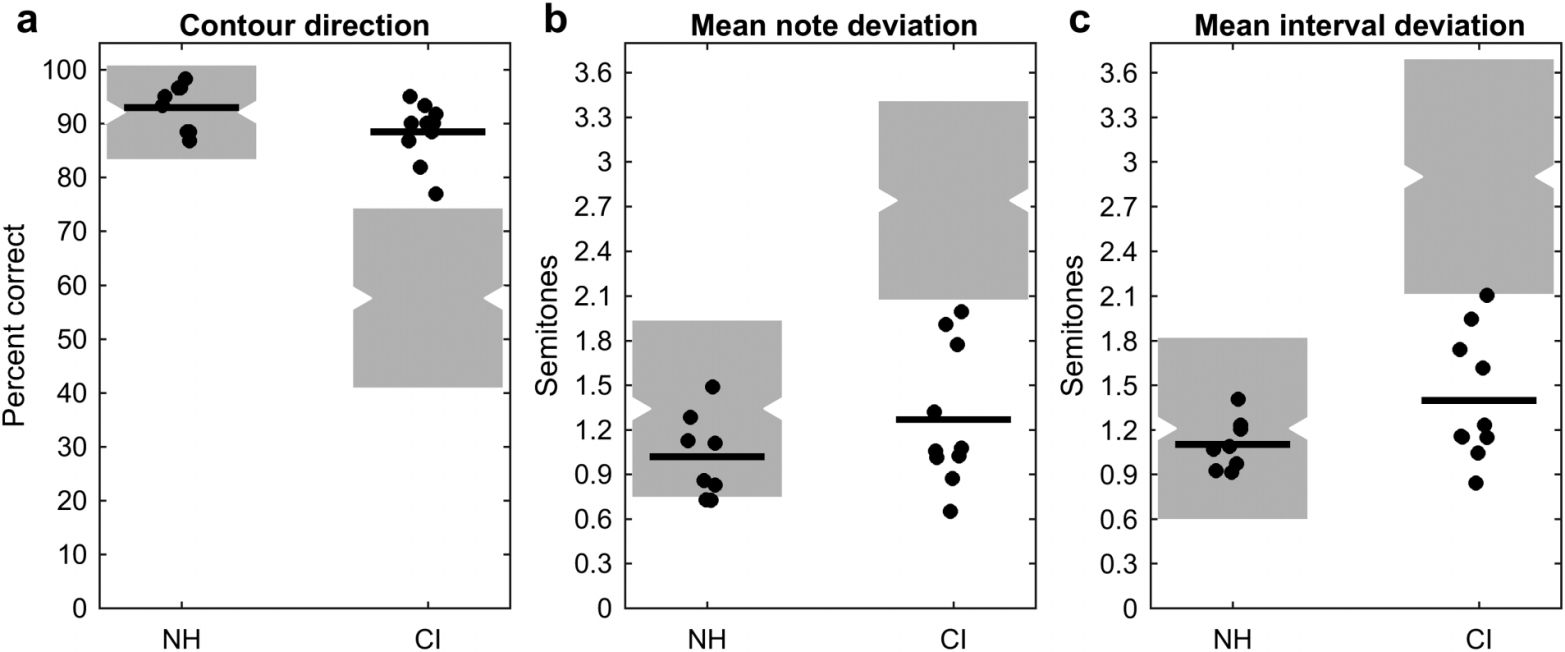
Singing accuracy in cochlear implant (CI) and normal hearing (NH) groups based on the three pitch-related metrics: (a) contour direction, (b) mean note deviation, and (c) mean interval deviation. For each panel, the data from the eight children with NH and the 10 Little Dolphins choir members with CIs are plotted in filled dots, and the mean values of the data are represented by the solid lines. The gray shades represent ± 1 *SD*, and the notches represent the mean values of the data from the two previous studies (Mao et al., 2013; Xu et al., 2009; NH, *n* = 51; CI, *n* = 44).

To quantify the singing accuracy, three pitch-based metrics developed in our previous studies (Mao et al., 2013; Xu et al., 2009) were used on the pitch contour data in this study. Figure 2 plots the results of the three pitch-related metrics of children with NH and children with CIs. The group mean and standard deviation of each of the metrics are provided in Table 2. Because the pretraining data from the choir members were unavailable, in order to compare the singing proficiency of the Little Dolphins and other children with CIs with no specific singing training, we added the data from 51 children with NH and 44 children with CIs from two previous studies (Mao et al., 2013; Xu et al., 2009) in Figure 2. Note that the three pitch-related metrics were based on the first 96 notes of the song *Little Carp Jumping the Dragon Gate* for the 10 Little Dolphins and eight controls with NH in this study. In the two previous studies, the same metrics were based on other children's songs (e.g., *Happy Birthday, Frère Jacques,* and *Twinkle Twinkle Little Star*) that have a simpler melodic structure and a fewer number of notes in comparison to the target song of this study.

**Table 2.** Mean and standard deviation of the five acoustic metrics in the children with normal hearing (NH) and cochlear implants (CI) of this study and the summary of independent-samples *t* test results between the two groups of children.

Acoustic metrics	NH		CI		*t*	*p*
	*M*	*SD*	*M*	*SD*		
Contour direction (%)	93.03	4.45	88.52	5.41	1.897	.076
Mean note deviation (semitone)	1.02	0.28	1.27	0.44	1.341	.199
Mean interval deviation (semitone)	1.10	0.17	1.40	0.40	1.859	.082
Mean duration ratio deviation	0.41	0.05	0.39	0.04	0.948	.357
Mean absolute duration deviation (s)	0.25	0.04	0.18	0.04	3.844	**.001**

*Note.* Bold = statistical significance of the t test result.

Figure 2a presents the percent correct of contour direction. If any two consecutive sung notes had the same ascending or descending pattern as the corresponding target notes, the contour direction was 100% correct. The chance performance was 50% correct. The mean score of this measure for the eight children with NH was 93.0% correct. The mean score for the 10 Little Dolphins children with CIs was 88.5% correct, slightly lower than the controls with NH but remarkably higher than the chance level of 50% correct. A two-sample *t* test revealed no significant difference between children with NH and children with CIs (*t* = 1.897, *p* = .076). Compared to the previous studies, the percent correct scores of contour direction of the eight subjects with NH in this study were similar to those of previously reported children with NH (Mao et al., 2013; Xu et al., 2009). A two-sample *t* test revealed no significant difference between the eight children with NH in this study and the 51 children with NH from the two previous studies (*t* = 0.275, *p* = .785). However, the percent correct scores of the 10 choir members with CIs were much higher than those of the previously reported 44 children with CIs who had a mean contour direction of only 57.7% correct. A two-sample *t* test showed a highly significant difference between the 10 Little Dolphins children with CIs and the 44 children with CIs from the two previous studies (*t* = 5.812, *p* < .0001).

Figures 2b and 2c plot the mean deviation of the normalized F0 across the notes and the mean deviation of the pitch intervals. These two measures represent the amount of pitch deviations in the sung notes relative to the target notes. As shown in Figure 2b, the mean note deviation for the eight children with NH and the 10 Little Dolphins choir members were on average 1.02 and 1.27 semitones, respectively. The two-sample *t* test revealed no statistical significance between these two groups (*t* = 1.341, *p* = .199). Likewise, as shown in Figure 2c, the eight children with NH had an average of 1.10 semitones in mean interval deviation, and the 10 children with CIs in the choir had an average of 1.40 semitones in that measure. There were no group differences as shown by the two-sample *t* test (*t* = 1.859, *p* = .082). These nonsignificant results indicated that the choir members with CIs, as a group, showed no difference from the children with NH on the two pitch-related measures. That is, the performance of vocal singing in these children with CIs was similar to the children with NH in terms of pitch accuracy. When compared with the combined larger samples of the two previous studies (Mao et al., 2013; Xu et al., 2009), the eight children with NH in this study showed no significant differences from the 51 children with NH reported in previous studies for the two measures (mean note deviation: *t* = 1.522, *p* = .133; mean interval deviation: *t* = 0.503, *p* = .617). However, the Little Dolphins choir members in this study showed highly significant differences from the children with CIs reported in previous studies for the two measures (mean note deviation: *t* = 6.661, *p* < .0001; mean interval deviation: *t* = 5.861, *p* < .0001). The averages of the mean note deviation and mean interval deviation in the previously reported 44 children with CIs were 2.74 and 2.90 semitones, respectively. These results were reminiscent of the mean pitch direction discrimination threshold of 3.0 semitones reported in Kang et al. (2009). Note that the pitch accuracy analyses in this study and the two previous studies were based on different songs. The target song in this study presents a greater pitch range and a more complex melodic structure than the songs used in the two previous studies. The significant difference between the Little Dolphins and the 44 children with CIs in the two previous studies suggested that the Little Dolphins with intensive music training showed much higher proficiency in singing than the children with CIs who received little or no music training.

**Figure 3.**
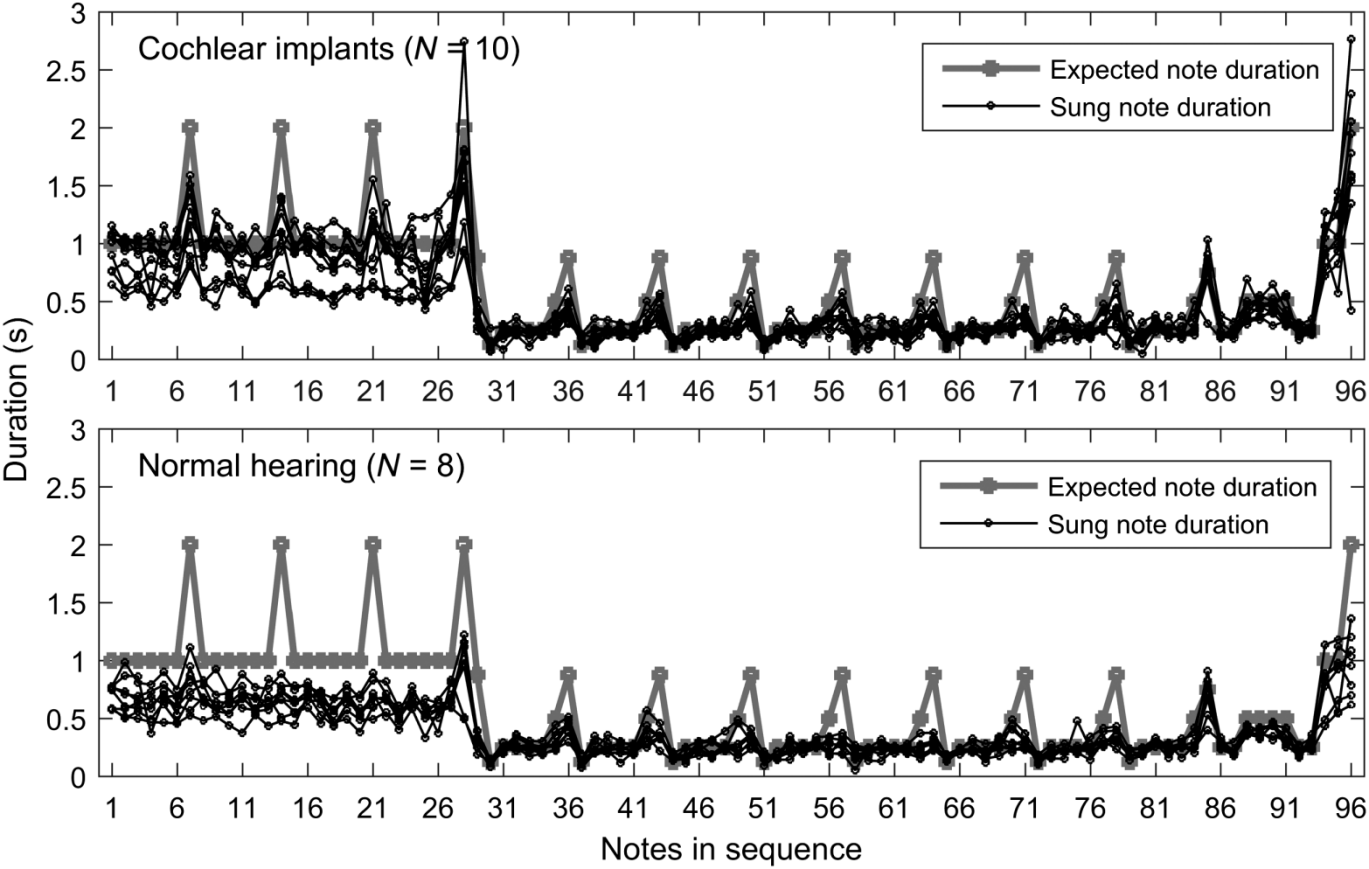
Duration of the first 96 notes in the song *Little Carp Jumping the Dragon Gate.* The top panel plots data from the 10 choir members with cochlear implants, and the bottom panel plots the data of the eight children with normal hearing. The thick gray line represents the target duration of the notes. The black lines represent the note duration produced by children with cochlear implants and children with normal hearing.

Figure 3 plots the duration of each note for the tested 96 notes in the song *Little Carp Jumping the Dragon Gate* for all subjects. All children singers (subjects with NH and CI) appeared to follow the target tempo but tended to sing too fast.

**Figure 4.**
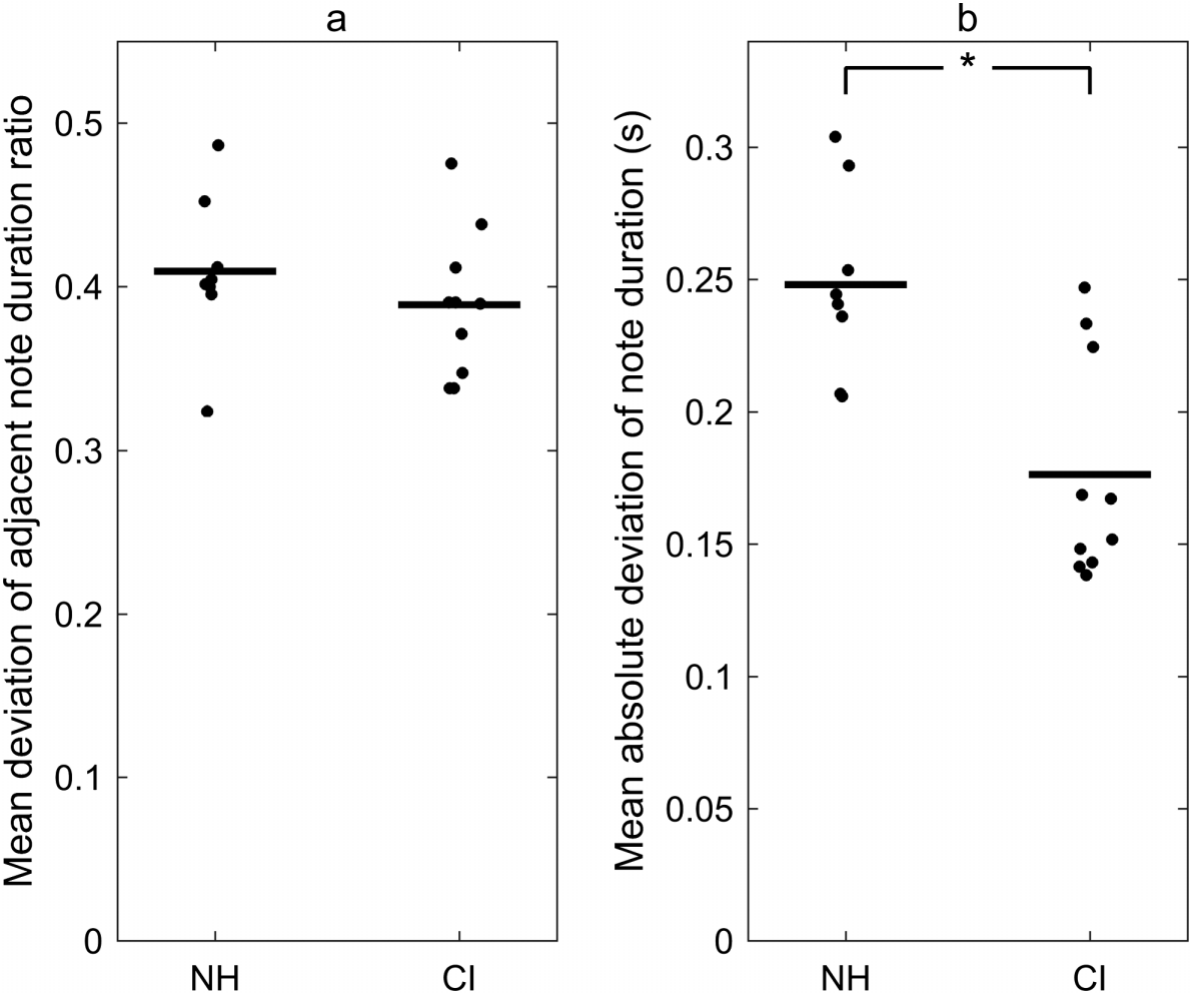
Tempo accuracy of the singing in cochlear implant (CI) and normal hearing (NH) groups. (a) Mean deviation of adjacent note duration ratio. (b) Mean absolute deviation of note duration. The asterisk indicates statistical significance (*p* < .05) of the difference between the NH and CI groups.

To quantify the accuracy in tempo of the singing, we derived two rhythm-based metrics, that is, mean deviation of adjacent note duration ratio and mean absolute deviation of note duration. A perfect score for both measures would be 0. The first measure evaluated the tempo of the singing in a relative term, and the second measure evaluated the tempo of the singing in an absolute term. The group mean and standard deviation of the two tempo-related metrics are provided in Table 2. As shown in Figure 4a, the eight children with NH and the 10 Little Dolphins children with CIs had similar relative tempo accuracy. A two-sample *t* test showed no significant difference in mean deviation of adjacent note duration ratio (*t* = 0.948, *p* = .357). On the other hand, as shown in Figure 4b, the Little Dolphins children with CIs had an average of 0.176 s deviation from the target notes, whereas the children with NH had an average of 0.248 s deviation from the target notes in the absolute duration. This observation indicated that the durational pattern of the singing by the choir members with CIs matched the target notes to a higher degree than that of the children with NH. The two-sample *t* test yielded a significant difference between the NH and CI groups (*t* = 3.844, *p* = .0014).

**Figure 5.**
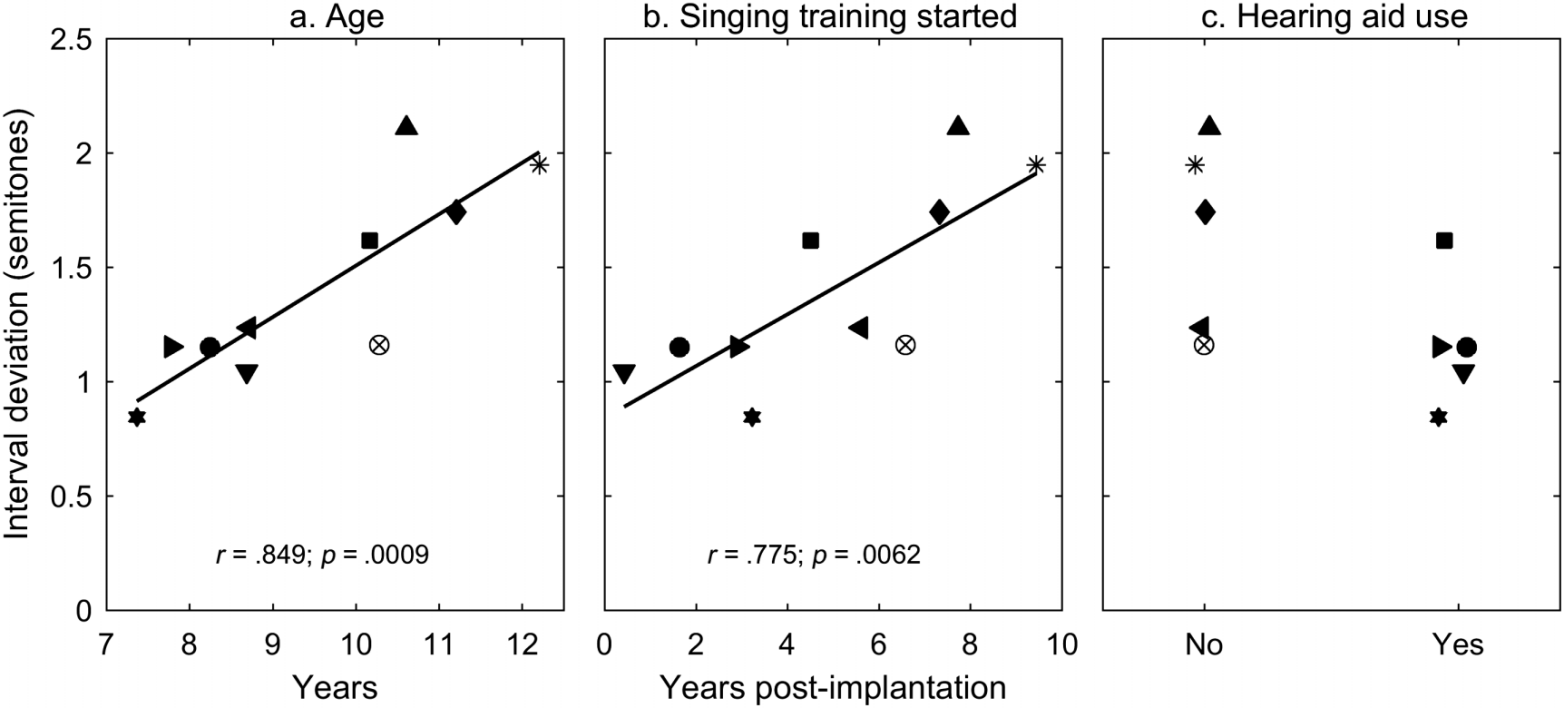
Relationship of pitch accuracy in singing and demographic factors of the Little Dolphins choir members with cochlear implants (CIs). (a) Mean interval deviation as a function of chronological age. (b) Mean interval deviation as a function of duration postimplantation before starting singing training. (c) Mean interval deviation in the children with CIs who used a hearing aid on the contralateral side and those who did not. In each panel, a different symbol represents a different subject (*N* = 10). The correlation coefficient (*r*) and the *p* value are provided at the bottom of a and b.

Although the Little Dolphins children with CIs sang on par with the children with NH, a fairly large amount of individual variability could be observed. We performed correlation analyses in an attempt to identify what demographic factors might account for the variability. Because the mean note deviation (see Figure 2b) and the mean interval deviation (see Figure 2c) were highly correlated with each other (*r* = .95) and both representative of the pitch accuracy of the singing, we chose the mean interval deviation as the outcome measure. Note that the mean deviation of pitch intervals defines the pitch difference of two adjacent notes in the sung notes relative to the target notes. The greater value indicates the poorer pitch performance. As shown in Figure 5, three factors appeared to be associated with the performance variability. Chronological age was positively correlated with the mean interval deviation (*r* = .849, *p* < .001; see Figure 5a). Thus, the younger members of the choir tended to sing more accurately in pitch. More interestingly, the duration postimplantation before joining the choir showed a positive correlation with the mean interval deviation (*r* = .775, *p* < .01; see Figure 5b). This suggested that the sooner the children started the singing training after implantation, the more accurate they could sing. In fact, the chronological age and the duration postimplantation before joining the choir showed a positive correlation (*r* = .837, *p* = .0013). Because the choir members were recruited around the same time, the younger children also started singing training at a younger age and had been implanted for a shorter period before the start of singing training than the older children. In addition, half of the children with CIs used an HA in the contralateral ear, and the other half did not. The best four performers used an HA in the contralateral ear, and the poorest three performers did not (see Figure 5c). It seemed that there was a tendency of better pitch performance with HA usage than without. Given the small sample size, a Mann–Whitney *U* test was conducted to test whether the children with CIs with an HA showed difference in pitch accuracy from those without an HA. The result supported the benefit of HA usage on pitch performance in children with CIs (*p* = .0159). Considering the very small sample size, the statistical test was less robust, and the result should be interpreted with caution.

## Discussion

Pitch information carries important linguistic and music functions. However, because of the inherent limitations of signal-processing strategies in CI technology and the damaged auditory periphery and central pathway in the listeners with hearing impairment, CI users usually demonstrate poor ability in accurately perceiving and producing pitch information in speech and music activities. Poor pitch and timbre representation in CIs might even hinder any efforts that utilize music training as a means of rehabilitation in CI users. Some recent studies on music training, however, reported the facilitating role of music training in improving CI users' perceptual ability in pitch-related tasks (J. K.-C. Chen et al., 2010; Cheng et al., 2018; Fu et al., 2015; Gfeller, 2016; Polonenko, Giannantonio, Papsin, Marsella, & Gordon, 2017; Torppa et al., 2014).

This study conducted comprehensive acoustic analyses to evaluate the accuracy of singing performance after a long period of intensive music training in prelingually deafened children with CIs. Our results revealed that the children with CIs showed patterns compatible with the target music score on all five measures, which covered both pitch-related and rhythm-related aspects of their singing performance. Compared to the children with NH in a similar age range, the children with CIs showed equally good singing ability with no significant difference from the children with NH on most metrics. For the only metric of the absolute deviation of note duration that showed significant differences between the two groups of children, the children with CIs in fact matched with the target tempo better than the children with NH did. In addition, the children with CIs in this study who received intensive formal musical training significantly outperformed the children with CIs in previous studies who received no specific music training in all pitch-based measures. Note that the songs sung by the children with CIs analyzed in the previous two studies were much simpler and shorter with moderate pitch range in comparison to the target song analyzed in this study. These results provide evidence that high singing proficiency in children with CIs is possible after rigorous music training.

Regardless of the increasing amount of research findings in favor of the beneficial impact of music training on pitch-related performance in speech and music in CI users (J. K.-C. Chen et al., 2010; Fu et al., 2015; Gfeller et al., 1998; Torppa et al., 2014; Torppa, Faulkner, Kujala, Huotilainen, & Lipsanen, 2018), the underlying mechanisms accounting for the performance gain remains unclear. With the advent of modern brain imaging technologies, more and more evidence has been reported to show experience-related plasticity in the human brain (Green & Bavelier, 2008; Kempermann et al., 2010; Limb, Molloy, Jiradejvong, & Braun, 2010). For children with congenital or prelingual deafness, although the auditory deprivation caused degeneration in central auditory system, which resulted in cascading adverse effects at multilevels along the auditory pathway (Hardie & Shepherd, 1999; Kral, Hartmann, Tillein, Heid, & Klinke, 2000; D. R. Moore, 1994; Nordeen, Killackey, & Kitzes, 1983; Ryugo, Rosenbaum, Kim, Niparko, & Saada, 1998), researchers found that the human central auditory system maintained maximal plasticity until approximately 4 years post the onset of deafness. After 7 years of sensory deprivation, the plasticity was greatly reduced (Sharma, Dorman, & Kral, 2005; Sharma, Dorman, & Spahr, 2002; Thai-Van et al., 2007). Eight of the children with CIs in this study received implantation before 4 years of age and the other two between 4 and 7 years of age (see Table 1). These children regained auditory stimulations, even though they were in the form of electrical pulses, within the sensitive period of auditory plasticity. Previous studies of auditory evoked potentials in children with CIs suggested that children with implant demonstrated normal-like development of the rostral auditory brainstem (Gordon, Papsin, & Harrison, 2006). One important finding of this study was that the singing performance was correlated with the duration between the implantation surgery and the beginning of music training. The shorter the duration, the better the singing performance of the choir members (see Figure 5b). This finding indicates that brain plasticity for musicality works the best when the brain is new to electrical stimulations.

Although the children with CIs in this study achieved high proficiency in singing, especially in the pitch-related measures as the children with NH, these two groups of children differed greatly in the training paradigms. The children with NH were casual singers who only had a 2-week training to learn the song. However, we should note that the children with NH had life long experiences with other songs and other music forms. Learning a new song can be viewed as the generalization of their overall music skills. The children with CIs, on the other hand, had no music experience before joining the choir and had gone through a much longer period of intensive training for 21 months. They practiced for a whole year to achieve satisfactory singing of the eight notes spanning one octave on a music scale. Our comparison between children with CIs and children with NH, albeit unbalanced in the length of training, further highlighted the challenges the children with CIs faced in vocal singing. As children with NH have intact instantaneous closed-loop auditory feedback system between hearing and vocalization, they can learn vocal control for accurate pitch production in an efficient and quick fashion. By contrast, the children with CIs could hardly use auditory feedback to effectively self-monitor or self-correct the laryngeal movement for pitch production. Instead, we assumed that they might rely more on somatosensory information to guide the laryngeal control for correct pitch production. Laryngeal somatosensory receptors provide important information regarding muscular adjustment and coordination for precise control of the frequency, timing, and intensity of vocal fold vibration (Gozaine & Clark, 2005; Sundberg, 1987; Wyke, 1974). Typically developing speakers use both auditory and somatosensory feedback to regulate the movement of vocal folds and relevant muscles (Larson, Altman, Liu, & Hain, 2008). However, the children with CIs might not be able to establish correct physiological–acoustic relationship for pitch based on their own auditory feedback. In this case, the instant feedback from the music instructors and their parents was likely to help them form the connection between the target pitch level and laryngeal muscular control. The somatosensory information associated with the pitch control could be used by the children with Cls to stabilize their voice control for different pitches.

When comparing the tempo of singing between the children with CIs and the children with NH, we found that both groups of children showed well-aligned patterns with the target notes of the musical score in terms of relative durations (see Figure 3). The children with CIs showed a better matched pattern on the absolute note duration to the target notes than the children with NH did. The children with NH sang too fast, especially for those longer notes. This is a common problem in casual singers in the general population (Dalla Bella et al., 2007). Because the CIs can deliver accurate rhythmic information to the users and the choir members with CIs had received rigorous training in singing and performed frequently on stage, it was not surprising that they followed the absolute tempo of the song better than the casual singers with NH.

Although the sample was very small, this study also revealed that the children with CIs who used an HA in the contralateral ear performed better in vocal singing than those who did not (see Figure 5c). Presumably, the bimodal users (i.e., with one CI and one HA) had some usable acoustic hearing on the HA side. The acoustic stimulation provided by HAs might have conveyed useful F0 information in comparison to the electrical stimulation provided by CIs. Previous research suggested that combined electrical–acoustical stimulation promoted the use of low-frequency residual hearing, and the adult CI recipients who used the bimodal hearing performed better on music perception (Arehart, Croghan, & Muralimanohar, 2014; Crew, Galvin, & Fu, 2016; Dorman, Gifford, Spahr, & McKarns, 2008; El Fata, James, Laborde, & Fraysse, 2009; Gfeller et al., 2012; Kong, Mullangi, & Marozeau, 2012; Looi, McDermott, McKay, & Hickson, 2008; Prentiss, Friedland, Nash, & Runge, 2015) or lexical tone perception (Li, Zhang, Galvin, & Fu, 2014) than the unilateral CI users. In prelingually deafened children with CIs, previous research revealed mixed results on the benefit of bimodal hearing for music or pitch perception (Bartov & Most, 2014; Hopyan, Peretz, Chan, Papsin, & Gordon, 2012; Looi & Radford, 2011; Polonenko et al., 2017). Our results, although small scale in data set, supported the idea that bimodal hearing was beneficial to the development of music ability in children with hearing impairment.

This study provided encouraging evidence that prelingually deafened children with CIs could achieve outstanding vocal singing proficiency. Because no perceptual data from the choir members with CIs were available in this study, it is unknown whether their superb vocal singing ability that resulted from the intensive music training would translate into better music perception, pitch perception, tone perception, speech perception, and eventually academic performance, and vice versa. Torppa et al. (2018) recently reported that children with CIs with regular informal singing at home outperformed children with CIs with no singing activity in speech-in-noise perception tasks. It is conceivable that intensive formal music training might also enhance the perceptual performance and other aspects of speech-language abilities in children with CIs. Furthermore, because of the absence of any longitudinal data in this study, the music development trajectory of the children with CIs was unknown. Finally, their rigorous training paradigm might be difficult to replicate for practical reasons. Would a modified version of training be just as efficient so that the success of Little Dolphins can be replicated in or generalized to a wider population? These are all important questions that the research community should address in the future.

## Conclusions

In summary, as the first attempt to evaluate the effects of music training on vocal singing ability in children with CIs, this study showed that the prelingually deafened children with CIs could achieve high proficiency in vocal singing following a long-term intensive formal music training. Multiple factors, including early start of music training after implantation and use of bimodal hearing, contributed to the development of music ability in children with CIs. The results of their high accuracy in vocal singing challenges our conventional, pessimistic view of poor performance in vocal singing in this population. This study has important implications in clinical practice. The positive outcome of singing ability in children with CIs advocated the use of music activities and music training in rehabilitation to improve the communicative abilities and self-esteem for deaf children with CIs. For future studies, a longitudinal investigation with a larger size of participants and better designed music training should be carried out to observe the impact of music training on the development of musicality in children with CIs. Last but not least, perceptual tests and speech-language tests should also be implemented on musically trained children with CIs and be compared with nonmusically trained children with CIs and NH. A number of recent studies reported positive results regarding the facilitating role of music training on speech and language abilities in children with NH (Degé & Schwarzer, 2011; Nan et al., 2018; Tierney, Krizman, & Kraus, 2015). This type of research will enable us to examine whether music training promotes the development of speech-language abilities and overall perceptual ability in children with CIs.
